# Apoptotic Induction and Anti-Migratory Effects of *Rhazya Stricta* Fruit Extracts on a Human Breast Cancer Cell Line

**DOI:** 10.3390/molecules24213968

**Published:** 2019-11-01

**Authors:** Mohammed Al-Zharani, Fahd A. Nasr, Nael Abutaha, Ali S. Alqahtani, Omar M. Noman, Mohammed Mubarak, Muhammad A. Wadaan

**Affiliations:** 1 Imam Mohammad Ibn Saud Islamic University (IMSIU), College of Science, Biology Department, Riyadh 11623, Saudi Arabia; MMyAlzahrani@Imamu.edu.sa; 2 Medicinal Aromatic, and Poisonous Plants Research Centre, College of Pharmacy, King Saud University, Riyadh 11451, Saudi Arabia, , onoman@ksu.edu.sa (O.M.N.); 3 Bioproducts Research Chair, Department of Zoology, College of Science, King Saud University, Riyadh 11451, Saudi Arabia; wadaan@ksu.edu.sa; 4 Electron Microscope Unit, King Saud University Medical City, Riyadh 11451, Saudi Arabia; mohammedahmed_62@yahoo.com

**Keywords:** *Rhazya stricta*, breast cancer, cell migration, apoptosis, GC-MS

## Abstract

*Rhazya stricta* is a medicinal plant that is widely used in Saudi folklore medicine for treatment of various diseases. *R. stricta* fruit powder was sequentially extracted with *n*-hexane, chloroform, ethyl acetate, and methanol using a Soxhlet extractor. The cytotoxic effects of these fractions on human breast cancer cells (MDA-MB-231 and MCF-7) and non-tumorigenic control cells (MCF-10A) were evaluated via cell viability measurements, microscopy, gene expression, and migration assays. Moreover, the effect of the most promising extract on 7,12-dimethyl-benz[a]anthracene (DMBA)-induced breast cancer was investigated in rats. The promising extract was also subjected to gas chromatography–mass spectrometry. Fruit extracts of *R. stricta* were significantly cytotoxic toward all tested cell lines, as demonstrated by MTT and LDH assays. Treatment of MDA-MB-231 cells with fruit ethyl acetate fraction (RSF EtOAc) increased expression 11of P53, Bax and activation of caspase 3/7. A cell migration scratch assay demonstrated that extracts at non-cytotoxic concentrations exerted a potent anti-migration activity against the highly invasive MDA-MB-231 cell line. Moreover, RT-PCR results showed that RSF EtOAc significantly downregulated MMP-2 and MMP-9 expression, which play an important role in breast cancer metastasis. Histological studies of breast tissue in experimental animals showed a slight improvement in tissue treated with fruit ethyl acetate extract. GC-MS chromatogram showed thirteen peaks with major constituents were camphor, trichosenic acid and guanidine. Our current study demonstrates that fruit extracts of *R. stricta* are cytotoxic toward breast cancer cell lines through apoptotic mechanisms.

## 1. Introduction

Globally, breast cancer is the second most common cancer after lung cancer. It is a major public health issue and is the leading cause of cancer death in women [1], with an estimated 522,000 deaths and 1.7 million new cases reported worldwide in 2012 alone [2]. In Saudi Arabia, breast cancer is the leading cause of cancer death [3] with an estimated 1856 new cases in 2014 according to the Saudi cancer incidence report [4]. Cancer cells can develop resistance to anticancer drugs through different mechanisms such as drug efflux, cell death inhibition, epithelial-mesenchymal transition, drug target alteration, DNA damage repair, and epigenetic modifications. More than 50% of patients experience cancer relapse and die from acquired resistance [5].

Many currently used anti-cancer drugs, such as the taxanes, become ineffective for breast cancer treatment because of acquired resistance to the treatment [6]. Therefore, this necessitates the search for new therapeutic agents to overcome acquired resistance in endocrine-resistant patients.

In recent years, use of plants in primary health care and phytotherapeutic research has increased owing to identification of bioactive molecules in medicinal plants and growing interest in alternative medicines. According to the World Health Organization (WHO), 80% of people rely on plant-based traditional medicines for primary health care [7]. Over 50% of anticancer drugs used in clinical trials have been isolated from plant-based natural sources [8].

*Rhazya stricta* Decne (locally known as harmal) is a medicinal plant belonging to the Apocynaceae family and has been traditionally used for treating various diseases in many Middle East and South Asian countries. *R. stricta* is used for treatment of various disorders such as diabetes, stomach disorders, and intestinal illness. It is also used as a purgative, antihelminthic, antipyretic, and anti-inflammatory medicine, and to treat sore throats and chronic rheumatism [9]. Many compounds isolated from *R. stricta* have proven to be antineoplastic (16-epi-(*Z*)-isositsirkine), antimicrobial (akuammidine, rhazimine, stemmadenine, tetrahydrosecaminediol) and cytotoxic (didemethoxycarbonyltetrahydrosecamine, sewarine, tetrahydrosecamine, tetrahydrosecaminediol diacetate, vallesiachotamine, and D,L-1-(oxo-3,4-thero-3,4,5-trihydroxy-1-pentyl)- β-carboline) [9].

Previously, several studies investigated anti-proliferation activity of *R. stricta* extracts against different cancer cells [10,11,12]. However, our study is the first to evaluate *R. stricta* fruit extracts in an animal model (in vivo) and investigate the anti-migratory activity against the highly aggressive, invasive, and triple-negative (ER, PR and HER2 negative) MDA-MB-231 breast cancer cells (in vitro). Moreover, the effects of different fractions of *R. stricta* from fruit part on cytotoxicity of non-metastatic MCF-7 breast cancer (ER/PR positive) cells, and non-tumorigenic MCF-10A cells were assessed.

## 2. Results

### 2.1. Cytotoxic Activity of Rhazya stricta Fruit Extracts Against Breast Human Cancer Cell Lines

**Figure 1:**
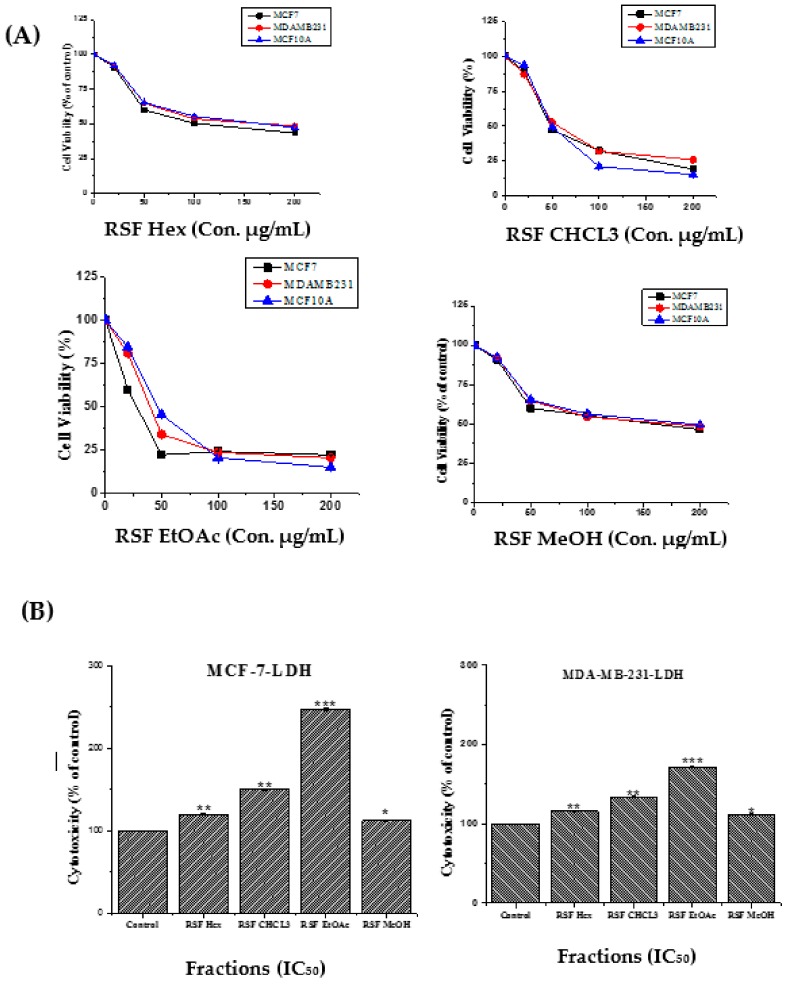
Cytotoxic effects of *R. stricta* Fruit fractions on human breast cancer cells. (**A**) Dose-dependent curves of different extract treatments. Cells were cultured in 24-well plates and treated with different concentrations (10–200 µg/mL) for 48 h. Cell viability was measured by MTT assay. (**B**) MCF-7, MDA-MB-231 and MCF-10A were treated at IC_50_ for 48h. LDH released into media was determined at 490 nm using a microplate reader. Statistical differences were analyzed using Student’s *t*-test. Data are presented as mean ± S.D. (*p < 0.05, **p < 0.01, ***p < 0.001 was considered significant compared to control) of three independent experiments.

Chloroform and ethyl acetate extracts displayed the strongest cytotoxicity against MCF-7 and MDA-MB-231 breast cancer cell lines compared to hexane and methanol extracts. The normal MCF-10A showed sensitivity to nearly all extracts. The fruit ethyl acetate (RSF EtOAc) and chloroform (RSF CHCL_3_) fractions inhibited MDA-MB-231 cells with IC_50_ values of 27 and 56 μg/mL, respectively. The two fractions also showed strong activity against MCF-7 cells (IC_50_ = 39 and 49 μg/mL, respectively). The other fractions exhibited different IC_50_ values (Table 1). Cell death after treatment was also determined by release of LDH into the incubation medium. Significant LDH release because of decreased membrane integrity in treated MCF-7 and MDA-MB-231 cells was observed after 48 h of treatment (Figure 1).

### 2.2. Apoptotic Activity of RSF EtOAc

The fruit ethyl acetate extracts (RSF EtOAc) of *R. stricta* exhibited the highest cytotoxic activity compared to other extracts at IC_50_. As such, this extract was further evaluated for apoptotic activity.

#### 2.2.1. Microscopic Studies

**Figure 2:**
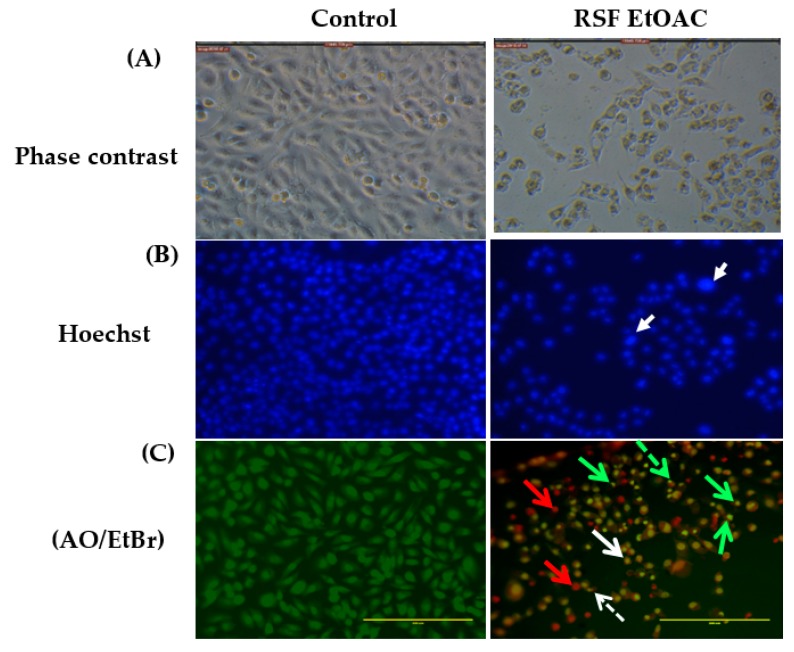
Detection of apoptosis in MDA-MB-231 cells after treatment with IC_50_ RSF EtOAC extract. (**A**) Phase-contrast microscopy showed a significant decrease in the number of cells after treatment versus untreated cells. (**B**) Nuclear morphology changes in both control and treated cells assessed by Hoechst staining, arrows indicating apoptotic cells (**C**) Viable cells show green fluorescence while necrotic and apoptotic cells show orange and yellow fluorescence. A dotted arrow indicates fragmented nuclei, a regular arrow indicates membrane blebbing, a green arrow represents apoptotic cells, and a red arrow represents necrotic cells.

Morphological changes of MCF-7 and MDA-MB-231 after 48 h of exposure to RSF EtOAc were observed using an inverted light microscope. A high-density monolayer of cells with intact membranes was observed in untreated cells. In contrast, treated cells showed reduced cell volume after treatment at IC_50_. Nuclear features of apoptosis were clearly observed after 48 h of treatment (Figure 2).

The results of acridine orange (AO) and ethidium bromide (EB) staining of MDA-MB-231 cells treated with RSF EtOAc extract are shown in Figure 2. AO is a fluorescent dye that stains DNA of live and dead cells, whereas EB is a fluorescent dye that stains DNA of cells that have lost membrane integrity [13].

After AO/EB staining viable cells were equally stained in green, early apoptotic cells were stained green/yellow, late apoptotic cells were stained yellow/orange with dots of condensed nuclei, and necrotic cells exhibited red fluorescent nuclei with no chromatin fragmentation (Figure 2).

#### 2.2.2. RT-PCR

**Figure 3:**
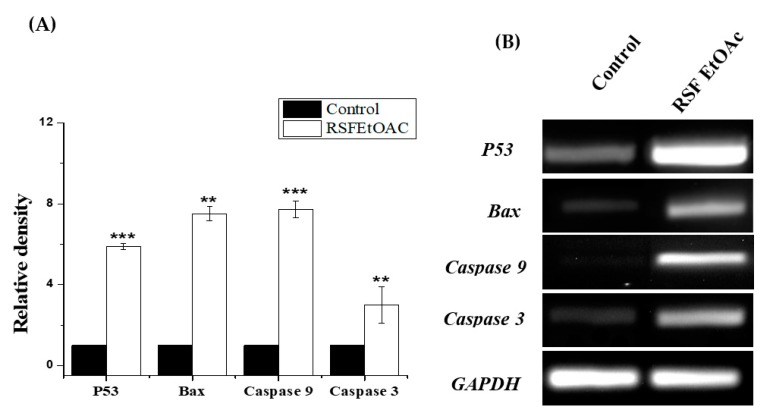
Effect of RSF EtOAc extract on *P53*, *Bax*, *caspase-9* and *caspase-3* mRNA levels in MDA-MB-231 cells. (**A**) The relative density variation of mRNA levels found in MDA-MB-231 treated at IC_50_ compared with control. (**B**) Ethidium bromide–agarose gel showing P53, Bax, and caspases 3 and 9 mRNA levels: Lane 1) Vehicle control, Lane 2) RSF EtOAc extract treated. Results are expressed as mean ± SD (n = 3). Statistical significance was assessed using Student’s t-test. Relative mRNA level was normalized to GAPDH mRNA level. *p < 0.05; **p < 0.01; ***p < 0.001 treatment group vs. control group.

To determine mRNA expression levels of apoptosis-related genes, we performed RT-PCR. After 24 h treatment of MDA-MB-231 cells with RSF EtOAc at IC_50_, p53, Bax, caspase 9, and caspase 3 mRNA band intensity increased 5.89-, 7.5-, 7.72-, and 3-fold, respectively (Figure 3). To confirm apoptosis, the commercial kit CellEvent™Caspase-3/7 detection was used to assess caspase-3 and -7 activity using fluorescence microscopy.

**Figure 4:**
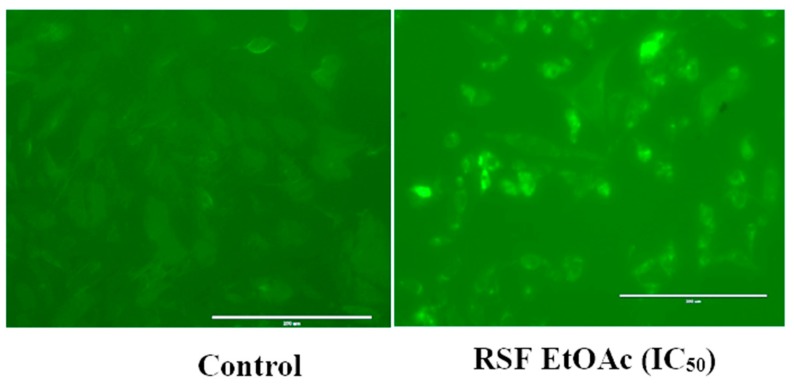
Activation of caspase-3 and 7 in MDA-MB-231 cells. Cells were treated with vehicle and at IC_50_ for 24 h. Activated caspase-3/7 was visualized by fluorescence microscopy. (RSF EtOAc) *R. stricta* fruit ethyl acetate extract was used.

As shown in Figure 4, MDA-MB-231 cells showed significant activation of caspase-3 and -7 after 24 h of treatment.

### 2.3. Effects of RSF EtOAc on Cell Migrations of MDA-MB-231

**Figure 5:**
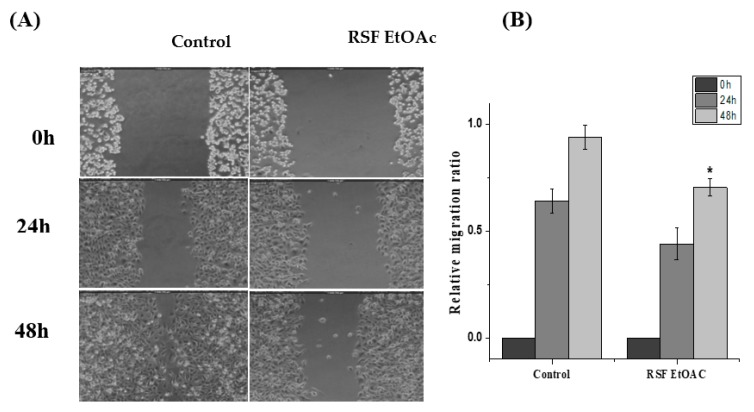
Effects of RSF EtOAC on MDA-MB-231 cell migration. (**A**) Images of the wounded monolayer of MDA-MB-231 cells captured immediately after wounding (t = 0 h) and following an incubation time of 24 h or 48 h. Cells were untreated (control) or treated at IC_25_. (**B**) Cell migration rate was calculated as described in materials and method. Experiments were performed in triplicate, *p < 0.05, **p < 0.01 vs. the control (Student’s two-tailed *t*-test).

To determine whether RSF EtOAC extract inhibits the migration of the highly metastatic cell, MDA-MB-231, in vitro wound-healing assay was performed. A non-cytotoxic concentration (1/2 IC_50_) of RSF EtOAc fraction was used to assess MDA-MB-231 migration. After 48 h, control MDA-MB-231 cells had migrated into the scratched area, whereas RSF EtOAc-treated cells migrated significantly less (*p* < 0.05, 48 h) (Figure 5).

**Figure 6:**
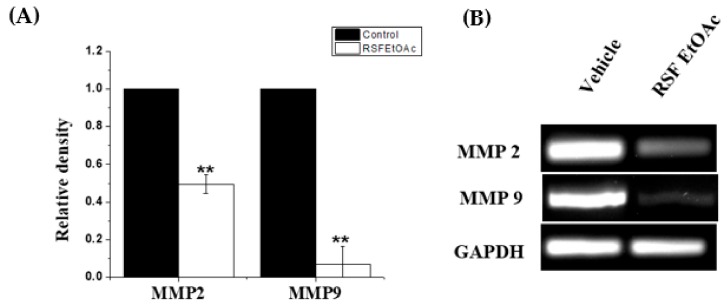
Effect of RSF EtOAc extract on expression of MMP-2 and MMP9 in MDA-MB-231 breast cancer cells. (**A**) The relative density variation of mRNA levels found in MDA-MB-231 treated at IC_50_ when compared with control. (**B**) Ethidium bromide–agarose gel showing MMP2 and MMP9 mRNA levels: Lane 1) Vehicle control, Lane 2) extract treated. Results are expressed as mean ± SD (*n* = 3). Statistical significance was assessed by Student’s *t*-test. Relative mRNA level was normalized to GAPDH mRNA level. *p < 0.05; **p < 0.01; treatment group vs. control group.

To explore the mechanism by which RSF EtOAC inhibits migration of MDA-MB-231 cells, RNA levels of matrix metalloproteinase (MMP-2 and -9) were evaluated by RT-PCR. Our results indicated that RSF EtOAc extract at IC_50_ suppressed mRNA expression of mRNA of MMP-2 and -9 compared to controls (Figure 6).

### 2.4. Histopathology and Morphological Observations

**Figure 7:**
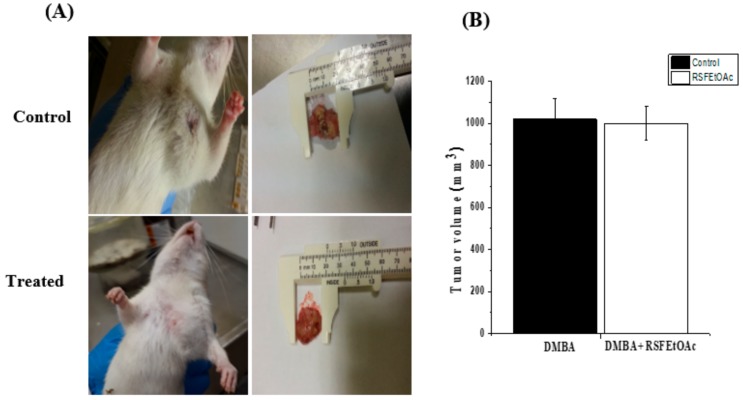
Breast cancer induced in Wistar albino rats (*Rattus norvegicus*) by subcutaneous injection of 9,10-dimethylbenz [a] anthracene (DMBA). (**A**) Tumor images in control (DMBA group) and after injection with RSF EtOAc extract and the size of excised tumors formed. (**B**) tumor volume in control and treated groups was calculated as described in materials and methods.

The fruit ethyl acetate extract (RSF EtOAc) of *R. stricta* was also chosen to evaluate in vivo in a DMBA-induced mammary tumorigenesis model. Immediately following DMBA injection animals were healthy and did not exhibit behavioral changes. Tumors were mostly observed after eight weeks. The tumor size was not significantly reduced after treatment with RSF EtOAc extract compared to control group. An average tumor volume of 1019 mm^3^ was calculated in control group, while upon RSF EtOAc extract injection, the average tumor volume was 1008 mm^3^ (Figure 7).

**Figure 8:**
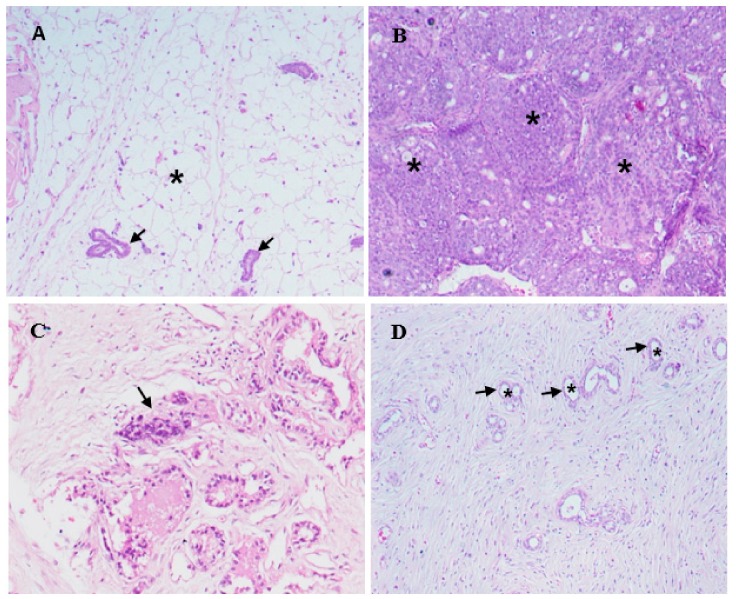
Light micrographs showing mammary gland tissues in control rats, DMBA-injected rats, and DMBA-injected rats treated with RSF EtOAc extract (**A**) Structure of mammary gland tissue in a control rat. Arrows indicate ducts and epithelial lining cells. Adipose tissue (*) surrounds the ducts (**B**) Mammary gland tissue of DMBA-injected rat showing markedly proliferating neoplastic ductal epithelial cells (*) obliterating the ductal lumina (**C**) Area of necrosis (arrow) in the mammary gland tissue of DMBA-injected rat, nuclear and cytoplasmic debris is seen in the area of necrosis (**D**) Mammary gland tissue of a DMBA-injected rat treated with RSF EtOAc extract. The ductal epithelial lining cells (arrows) are not proliferated and ductal lumina (*) are patent.

Histological examination of mammary gland tissue of control rats showed normal morphology of the ductular structures, which were surrounded by a small amount of fibrous connective tissue and adipose tissue (Figure 8A).

In mammary gland tissues of DMBA-injected rats, the ductal epithelial lining cells showed neoplastic changes identified as ductal carcinoma. Lining epithelial cells had markedly proliferated and appeared as densely packed cell layers that caused narrowing and even obstruction of the ductal lumina (Figure 8B). The proliferated neoplastic cells had pleomorphic nuclei varying in shape and size and had prominent nucleoli. Ductal cellular proliferation was so extensive in some areas of the mammary gland tissue that the histological architecture and organization was obscured. Large numbers of neoplastic cells were also found infiltrating the interstitial tissue. Scattered areas of necrosis of various sizes were observed, and the necrotizing process mainly involved the ductal elements of the mammary gland tissue (Figure 8C). No comparable histological changes were noticed in DMBA-injected rats that were treated with RSF EtOAc extract. Mammary gland tissue in this group showed no recognizable neoplastic proliferating cells in most of the examined ductal structures. In addition, no noticeable areas of necrosis were detected in the examined tissues of rats in this group (Figure 8).

### 2.5. Quantitative Phytochemical of R. stricta Fruit

Total phenol and flavonoid contents of *R. stricta* fruit fractions were quantified using gallic acid and quercetin standard curves (the standard curve equation: y = 0.0005 × x + 0.0505, R^2^ = 0.997 and y = 0.0052 × x + 0.1576, r2 = 0.996 respectively). Total phenol concentration is reported in µg/mL of extract, with results ranging from 220 to 257 µg/mL (Table 2). The concentration of flavonoids in fractions ranged from 40 to 75 µg/mL. RSF EtOAc fraction had the highest phenol and flavonoid content (Table 2). Antioxidant activities of fractions are represented in terms of percentage of scavenging activity of DPPH (%). The strongest antioxidant activity was observed in RSF EtOAc with a value of 51.81% (Table 2).

### 2.6. Gas Chromatography Mass Spectroscopy

**Figure 9:**
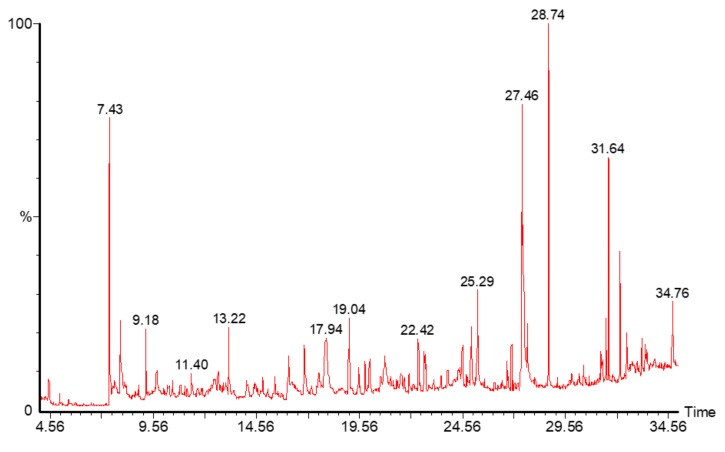
GC-MS analysis of phytochemical compounds in RSF EtOAc extract.

GC-MS chromatogram analysis of the RSF EtOAc fraction showed thirteen peaks (Figure 9) which showing the presence of thirteen compounds (Table 3). 

**Figure 10:**
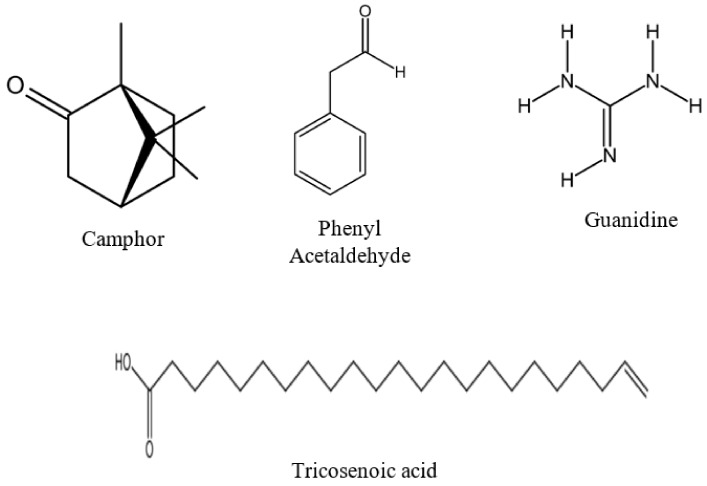
Major constituents in RSF EtOAc extract.

The GC-MS chromatogram and peak area of separation of the components are shown in (Figure 9). The major compounds were camphor (21%), trichosenic acid (15%), guanidine (14%) and phenyl acetaldehyde (8%) (Figure 10).

## 3. Discussion

In this study, fractions of *R. stricta* fruit were screened for their ability to induce cytotoxicity, apoptosis, and anti-migration activity in estrogen-positive (MCF-7), estrogen-negative (MDA-MB-231), and normal (MCF-10A) human breast cancer cell lines. To obtain a maximum amount and diversity of biologically active phytochemicals, we performed these extractions using solvents with varying polarity. All extracts exhibited anti-proliferative activity in a dose-dependent manner. However, our results revealed that the most promising extract was *R. stricta* fruit ethyl acetate extract (RSF EtOAc), which had an IC_50_ value 27 µg/mL in the MDA-MB-231 cell line. According to the American National Cancer Institute the IC_50_ value of a crude extract is considered promising if it is lower than 30 μg/mL [14]. RSF EtOAc fraction may be promising for use as anticancer treatments. Our results were consistent with the findings of Baeshen et al. [10], who reported that aqueous and ethanol extracts of *R. stricta* had anti-proliferative effects in both MCF-7 and MDA-MB-231 cells. Comparing IC_50_ values, our results showed higher activity of the extracts compared to their results, which may be attributed to the type of solvents and methods of extractions used.

The *R. stricta* was also cytotoxic to the MCF-10A cells (non-tumorigenic cells) which indicates the non-selective inhibition of cancer cell growth however, further investigation on fractionation of the extract might improve the cytotoxicity and selectivity.

The ability of RSF EtOAc extract to induce cell death was examined to clarify the mechanism by which these extracts inhibited cell growth. The inhibitory activity of *R. stricta* fractions on cell growth was associated with induction of apoptosis since treated MCF-7 and MDA-MB-231 cells exhibited the morphological features of apoptosis [15] such as viability loss, cell shrinkage, cell detachment, and nuclear condensation in treated cells (Figure 2).

Treatment of MDA-MB-231 with RSF EtOAc resulted in a significant increase in levels of p53 (Figure 3), a key tumor suppressor/regulatory gene in apoptosis induction following DNA damage caused by anti-cancer agents [16,17], resulting in growth arrest of cells in G1 phase or apoptosis [18,19]. Furthermore, caspase-3/7 activities were examined by CellEvent caspase-3/7 green detection and confirmed the above results (Figure 4). Caspase 3/7 participate to the majority of steps which takes place during apoptosis. Here, activation caspase 3/7 indicated that caspases act as key regulators in RSF EtOAc extract -induced apoptosis. Our results agree with the findings of Baschen et al. that ethanolic extracts of *R. stricta* induce apoptosis through upregulation of Bax proteins [10]. A recent study reported a similar effect [11] when evaluating crude alkaloid extracts of *R. stricta* leaves on human lung cancer cells A549. More than 90% of deaths from cancer occur due to metastasis of cancer cells, which remains one of the biggest challenges in cancer treatment [20,21]. Metastasis is considered the main cause of ineffectiveness of chemotherapeutic treatment and subsequent cancer deaths. However, prevention of metastasis improves chances of survival. Therefore, we examined the influence of plant extracts on the migratory capacity of the highly invasive MDA-MB-231 breast cancer cell line. The in vitro wound healing migration assay was used to survey the effects of selected extracts. Few studies have detailed anti-migration activity plant extracts. In this study, we showed for the first time that RSF EtOAc extracts have the ability to control MDA-MB-231 breast cancer cell migration at non-cytotoxic concentrations (Figure 5**)**. To evaluate the mechanism of the anti-metastatic effect, we examined *MMP-2* and *MMP-9* gene expression. Previous reports showed that MMPs are important in facilitating tumor progression, metastasis, invasion, and angiogenesis [22,23]. Expression of metalloproteinase plays a vital role in invasion of malignant cancer cells into normal tissue and reduces effectiveness of chemotherapy. Therefore, inhibition of MMP expression is a useful strategy for controlling cell migration [24,25]. We focused on evaluating MMP-2/-9 expression because they degrade the main component of the basement membrane. Previous studies have found that certain natural products can inhibit cancer metastasis by inhibition of ECM degradation through inhibition of matrix metalloproteinase [26,27,28,29,30]. In the present study, we have demonstrated for the first time that RSF EtOAc extract displayed a remarkable ability to inhibit metastasis via down regulation of *MMP-2* and *MMP-9* in MDA-MB-231 breast cancer cells (Figure 6). Our data indicated that RSF EtOAc might be a promising candidate for restricting growth of breast cancer cells. However, with the discovery of new potential therapeutic agents, breast cancer models are needed for preclinical trials. Therefore, in vivo models of human breast cancer are an indispensable tool in the development of new cancer therapeutics. There are several techniques to induce mammary tumors in rats such as genetic engineering, xenograft models, and chemical methods. The chemical method using DMBA is considered the most common method for use in preclinical studies and is a useful tool for study of cancer [31]. Our study is the first to evaluate *R. stricta* extracts (RSF EtOAc) using an animal model. In this study, tumors were observed in the 8^th^ week, in agreement with studies that reported that DMBA administration to female rats caused primary breast tumors within 2 to 3 months [32,33]. The histopathology of the tissue examined revealed little improvement in the tissue when compared to control. The only improvement seen was reduction in necrotic areas of the studied tissue (Figure 8). However, this result did not agree with cell culture (in vitro) results, although cell culture methods are the most used in preclinical studies. This may be due to animal tissues (in vivo) having stromal and 3D structures, which are not present in cell culture models [34].

Several reports on GC–MS analyses of plant extracts revealed that most of the plant extracts contained some of the phytocompounds or analogs of the phytocompounds present in *R. stricta*. Camphor, which is found in *Cinnamomum camphora* [35] and some other plants [36], was one of the major components in *R. stricta* (Table 3). It was traditionally used as perfumes, cosmetics, Food flavorings, fumigants, household cleaners, and analgesics [37]. It shows several biological activities, such as antimicrobial, antiviral, antitussive, and analgesic activities [38]. Regarding its cytotoxic effects, Camphor has been reported to be active against several cancer cell lines, including human lung cancer (A549), colon adenocarcinoma (DLD-1), human keratinocytes (HaCaT), and Human skin fibroblasts (WS1) cell lines [36]. Another major phytochemical, namely D-allose (7.2%) was also reported for its cytotoxic effects via inhibition of cancer cell growth [39]. Guanidine isolated from Polynesian *Monanchora* exhibited cytotoxic efficacy against human colorectal carcinoma (HCT116), promyelocytic leukemia (HL-60), and human lung normal (MRC5) cancer cells [40]. Moreover, aspidospermine and quebrachamine were reported to block the contractions of human prostatic tissue, cavernosum and guinea pig vas deferens and rabbit corpus spongiosum [41].

## 4. Materials and Methods

### 4.1. Plant Sample Collection and Extract Preparation

The fruits of *R. stricta* were collected from Riyadh, Saudi Arabia. The plant was identified by Dr. Jacob Thomas Pandalayil, Department of Botany and Microbiology, College of Science, King Saud University, and a voucher specimen (herbarium no. NATKSU-108) was deposited at the Department herbarium. *R. stricta* fruit were washed, dried, ground to powder, and sequentially extracted with solvents of decreasing lipophilicity (400 mL of *n*-hexane, chloroform, ethyl acetate, and methanol) using a Soxhlet extractor. The collected extracts were evaporated using a rotary evaporator under vacuum at 45 °C. The crude extracts were weighed and stored at –80 °C for further studies.

### 4.2. Cytotoxicity Assays

Determination of cytotoxicity (after a 48 h exposure period) was carried out by two following colorimetric methods. 

#### 4.2.1. MTT Assay

MCF-7 and MDA-MB-468 cells were cultured in DMEM (Gibco, ThermoFisher Scientific, CA, USA), and MCF-10A cells (were cultured in DMEM/F12 each containing 10% *v*/*v* FBS. Cells were seeded into 24-well cell culture plates at a density of 5 × 10^4^ cells per well in 1-mL aliquots of medium. Cells were permitted to attach for a period of 24 h at 37 °C and 5% CO_2_ in an incubator. Cells were treated for 48 h with *R. stricta* fruit fractions and 100 μL of MTT reagent (Invitrogen Life Technologies, CA, USA) (5 mg/mL in PBS) was added to all wells. Plates were incubated at 37 °C for 2–4 h. One milliliter per well of isopropanol-HCL was added to dissolve crystalline formazan. Reduced MTT formation was measured at 540 nm using a microplate reader (Thermo Fisher Scientific, Waltham, MA, USA). Wells with untreated cells were considered as controls. For each extract tested, IC_50_ (concentration of tested compound needed to inhibit cell growth by 50%) and cell viability was calculated using the following equation:

Cell Viability (%) = (O.D of treated sample)/(O.D of untreated sample) × 100%(1)

#### 4.2.2. Lactate Dehydrogenase (LDH) Cytotoxicity Assay

For each experiment, a fresh LDH mixture was prepared according to manufacturer instructions (LDH kit, Sigma-Aldrich, St. Louis, MO, USA). Cells were seeded in 24-well culture plates at a density of 5 × 10^4^ cells/well and allowed to grow for 24 h before treatment. Cells were treated at IC_50_ for 48 h. The supernatant (100 μL) was transferred to a new 96 well plate and mixed with 100 μL of LDH assay mixture. The reaction was incubated for 30 min at room temperature (25 °C) in the dark and analyzed using a microplate reader (Thermo Fisher Scientific) at 490 nm. The cytotoxicity of each fraction was calculated:

(%) = (O.D of treated sample)/(O.D of untreated sample) × 100%(2)

### 4.3. Assessment of Morphology of Apoptotic Cells by Phase-Contrast Inverted Microscopy, Fluorescent Hoechst 33,258 Staining, and Acridine Orange Ethidium Bromide Dual Staining

MDA-MB-231 cells were grown in 12-well plates and incubated for 48 h with and without RSF EtOAc extract at IC_50_. Morphological changes characteristic of apoptotic cells were observed using phase contrast inverted microscopy (MC-170 HD camera, Leica, Wetzlar, Germany) at 200× magnification. For Hoechst 33,258 staining, cells were treated then washed twice with PBS at room temperature, fixed with 4% paraformaldehyde, permeabilized using cold methanol, and stained with Hoechst 33,258 (Sigma) diluted in PBS (final concentration 0.5 µg/mL) for 30 min in the dark. Cells were examined for nuclear changes (i.e., chromatin condensation and nuclear fragmentation) using a fluorescence microscope attached to an Axiocam 506 color camera (Zeiss, Wetzlar, Germany).

For acridine orange/ethidium bromide dual staining, MDA-MB-231 cells were treated at IC_50_ for 48 h, directly stained with AO/EB (4 μg/mL) for 5 min, and imaged immediately using fluorescence microscopy (EVOS, Carlsbad, CA, USA).

### 4.4. Gene Expression Detection Using RT-PCR

MDA-MB-231 cells (5 × 10^4^ cells/well) were cultured in 6-well culture plates and treated with RSF EtOAc at IC_50_ for 24 h. Following incubation, total RNA was prepared using TRIzol (TRI Reagent) (Invitrogen; Thermo Fisher Scientific, Inc., Carlsbad, CA, USA), and cDNA synthesized with oligo dt primer using superscript II reverse transcriptase cDNA synthesis Kit (Invitrogen; Thermo Fisher Scientific, Inc., Carlsbad, CA, USA) according to the manufacturer’s instructions. PCR was performed using a Roter Gene machine, (QIAGEN, Hilden, Germany) with genes specific primers. Amplification products obtained by PCR were separated on 1.5% agarose gel, stained with ethidium bromide (0.5 μg/mL) and visualized under gel documentation system (Analytik Jena, Jena, Germany).

### 4.5. Caspase 3/7 Green Fluorescence Detection

MDA-MB-231 cells were seeded in 12-well culture plates at a density of 5 × 10^4^ cells/well and allowed to grow for 24 h before treatment. After IC_50_ treatment with and without the extract, cells were labeled with caspase-3/7 green detection reagent (5 μM) in DMEM medium in the dark at 37 °C for 30 min. Images were captured using fluorescence microscopy (EVOS, Carlsbad, CA, USA).

### 4.6. Scratch Wound Healing Migration Assay

MDA-MB-231 cells were seeded onto a 12-well tissue culture plate and grown to ~70–80% confluency. The monolayer was gently and slowly scratched with a sterile pipette tip (10 μL) across the center of the well. Medium was aspirated and washed twice with PBS to remove the detached cells. Fresh medium was added. Non-cytotoxic concentrations (1/2 IC_50_) of RSF EtOAc extract were added, and images were captured over several hours (0 h, 24 h and 48 h) to monitor closure or migration of cells to fill the scraped area using a phase contrast inverted microscope attached to a Leica MC-170 HD camera (Leica, Wetzlar, Germany). ImageJ software (NIH, Bethesda, MD, USA) was used to analyze the images. The relative migration ratio was calculated according to the following equation [42]:

Relative Migration Ratio = (Distance at 0 h-Distance at 48 h)/(Distance at 0 h)(3)

### 4.7. In Vivo Experimental Studies

Thirty female Albino rats (115 ± 15 g body weight, 49–59 days old) were obtained from the College of Pharmacy, King Saud University. The animals (five rats per cage) were housed in large cages at 25 ± 2 °C with a 12 h light/dark cycle in the animal facility of the Zoology Department, College of Science. Rats had free access to water and commercial pelleted diet (Saudi Grains Organization, Riyadh, Saudi Arabia). All procedures in this study were performed according to the Animal Ethics Committee, King Saud University, Zoology Department (KSU/Animal Ethics Approval/2018/(03) (2448)).

#### Tumor Induction and Plant Extract Treatment

Rats were divided into three groups of five rats each: Normal control (group I) and groups II and III where carcinoma was induced in the breast with a single subcutaneous dose on the right side. Twenty five milligrams of 7,12-dimethylbenz(a)anthracene (DMBA) was dissolved in 1 mL of sunflower oil and administered to each rat [43]. Animals were observed daily for general health. When mammary tumors appeared, group II (DMBA control group) received 100 µL of sunflower oil. Group III (DMBA + Extract) animals were injected with the extract (500 µg per rat, prepared in 100 µL of sunflower oil) directly into the tumor every 48 h for two months [44,45]. At the end of the treatment period, rats were fasted overnight and sacrificed by cervical dislocation under anesthesia using diethyl ether. A caliper was used to measure the tumor size and tumor volume was calculated using V = 0.5 × (a(b)^2^) formula, where “a” and “b” are the major and minor diameters of tumors, respectively [32]. Removed tumors were washed in ice-cold PBS (pH = 7.4) and weighed. Tumors were fixed in 10% buffered formalin solution and embedded in paraffin. The blocks were sectioned (5 µm), and slides were prepared with hematoxylin-eosin stain and examined using a compound microscope. 

### 4.8. Determination of Total Phenolic, Flavonoid Contents 

Total phenolic content of all fractions were measured according to the Folin-Ciocalteu method [46], with slight modifications. Briefly, 12.5 µL of extract (1 mg/mL prepared in methanol) was mixed with 125 µL of 25% Folin–Ciocalteu reagent in 96-well microplates and incubated for 5 min. Following incubation, 12.5 µL of 7% Na_2_CO_3_ was added, and the plate was mixed and in the dark for 1.5 h. Wells were read at 760 nm using microplate reader (Thermo Fisher Scientific, Waltham, MA, USA). Total phenolic content was quantified using a gallic acid standard curve. Total flavonoid content was calculated using aluminum chloride colorimetric assay [46]. In brief, 100 µL of each crude extract (1 mg/mL) and 100 µL of aluminum chloride (2%) were mixed together in a 96-well plate. The plate was incubated for 10 min (25 °C) and the absorbance was measured at 368 nm. A calibration curve of quercetin, a standard flavonoid, was used for estimation of flavonoids in the samples.

### 4.9. Antioxidant Activity using DPPH Radical Scavenging Method

The free radical scavenging activity of extracts was determined using 2,2-diphenyl-1-picrylhydrazyl (DPPH) [46]. Twenty microliters of each extract (1 mg/mL) was mixed with 80 µL of a methanolic solution of DPPH (100 mM) in 96 well plates. The plate was incubated in the dark for 30 min at 25 °C. Changes in absorption were read at 517 nm and radical scavenging activity was calculated by the following formula:

(4) $$\%\;\mathrm{Scavenging}\;=\;\frac{A517\;\mathrm{Control}-A517\;\mathrm{Test}}{A517\;\mathrm{Control}}\;\times 100$$

### 4.10. Gas Chromatography-Mass Spectroscopy (GC-MS)

GC-MS analysis was carried out using a Perkin Elmer Clarus 600 gas chromatograph/mass spectrometer (Turbomass, PerkinElmer, Inc., Waltham, MA, USA). An aliquot of 1 µL of the RSF EtoAc fraction was injected into the Elite-5MS column (30 m, 0.25 µm thickness, 0.25 µm internal diameter). The temperature programme started at 40 °C, was held for 2 min, then raised to 200 °C at a rate of 5 °C min^−1^ and held for 2 min. From 200 °C, the temperature was raised to 300 °C at 5 °C min^−1^ and held for 2 min. The carrier (helium gas) flow was set at 1.0 mL × min^−1^. The results were compared by using the National Institute of Standard and Technology (NIST) and WILEY Spectral libraries.

### 4.11. Statistical Analysis

Statistical differences between control and treatment groups were analyzed using Student’s *t*-test. Data are represented as the mean ± S.D. All statistical analysis and charts were generated using Origin Lab software version 8 OriginPro 8.0 software (OriginLab®, Northampton, MA, USA). Cell migration data were analyzed with ImageJ imaging software (NIH, Bethesda, MD, USA).

## 5. Conclusions

The present study revealed that *R. stricta* contains variety of secondary metabolites that possess anticancer potential based on the assays carried out. Thus, our study proposes that fruit extract of *R. stricta* containing bioactive compounds may possibly be used as a therapeutical source for discovery of drugs to manage cancer diseases. The present study thus paves the way for additional research on the isolation and characterization of the active fraction(s) of *R. stricta* to increase its cytotoxicity and selectivity.

## Figures and Tables

**Table 1:** Comparison of IC_50_ values for the different *R. stricta* fruit fractions towards MCF-7, MDA-MB-231 and MCF-10A breast cancer cell lines.

Fraction Abbrev.	Cell Lines and IC_50_ (µg/mL)		
	MCF-7	MDA-MB-231	MCF-10A
**RSF Hex**	104 ± 2.1	167 ± 1.4	168 ± 1.4
**RSF CHCL_3_**	49 ± 1.1	56 ± 0.6	49 ± 1.1
**RSF EtOAc**	39 ± 0.8	27 ± 0.5	47 ± 0.9
**RSF MeOH**	160 ± 1.5	177 ± 2.2	193 ± 0.6

**Table 2:** Phenolic, flavonoid contents and radical scavenging activity of *R. stricta* fruit fractions.

Fraction	Total Phenolics (µg/g)	Total Flavonoid (µg/g)	Antioxidant (%)
**RSF Hex**	235 ± 2.5	55.63 ± 1.1	44.25 ± 1.8
**RSF CHCL_3_**	246.67 ± 3.4	47.89 ± 2.3	37.66 ± 2.5
**RSF EtOAc**	257 ± 1.5	75 ± 2.5	51.81 ± 1.6
**RSF MeOH**	220 ± 1.7	40 ± 1.5	41.41 ± 2.1

**Table 3:** Compounds identified in the ethyl acetate fraction of *R. stricta* using GC-MS.

Compound Name	Chemical Formula	Molecular Weight (g/mol)	RT (min)	Area	Area %
**Phenylacetaldehyde**	C_8_H_8_O	120.15	9.18	257603	8.040
**(Dimethylamino)methylene malononitrile**	C_6_H_7_N_3_	121.140	9.70	37370	1.170
***trans*-2-Undecenal**	C_11_H_20_O	168.28	13.22	103901	3.240
**Dihydrocitronellal**	C_10_H_20_O	156.27	14.10	64583	2.010
**Linalyl butyrate**	C_14_H_24_O_2_	224.344	16.90	136684	4.260
**D-Allose**	C_6_H_12_O_6_	180.156	17.95	230986	7.200
**1-(3,4-Dimethoxyphenyl) ethanone**	C_10_H_12_O_3_	180.203	19.06	178135	5.560
**2,2-Tricosenoic acid**	C_23_H_44_O_2_	352.603	27.46	484030	15.100
**9-Octadecenoic acid**	C_18_H_34_O_2_	282.468	27.70	64120	2.000
**Aspidospermine**	C_22_H_30_N_2_O_2_	354.494	28.74	240055	7.490
**Quebrachamine**	C_19_H_26_N_2_	282.431	31.64	211407	6.590
**Camphor**	C_10_H_16_O	152.23	32.19	675960	21.080
**Guanidine**	CH_5_N_3_	59.07	34.76	465365	14.520
